# Supplementary material for: Optimizing the Preparation of Silk Fibroin Nanoparticles and Their Loading with Polyphenols: Towards a More Efficient Anti-Inflammatory Effect on Macrophages
Source: Pharmaceutics. 2023 Jan 12;15(1):263. doi: 10.3390/pharmaceutics15010263 (PMC9861267; doi:10.3390/pharmaceutics15010263)
Supplement: Supplementary file 1 [file pharmaceutics-15-00263-s001.zip › pharmaceutics-2094464_Supp Info.pdf]

pharmaceutics

Supplementary information for

Optimizing the preparation of silk fibroin nanoparticles and their loading with polyphenols: towards a more efficient anti-inflammatory effect on macrophages

Antonio José Ruiz-Alcaraz [1,†], María Ángeles Núñez-Sánchez [2,†], María Alejandra Asensio Ruiz [3], María Antonia Martínez-Sánchez [1,2], Alba Oliva-Bolarín [2], Teresa Martínez Martínez [3], José Julián Pérez Cuadrado [3], Bruno Ramos-Molina [2,*] and Antonio Abel Lozano-Pérez [3,4,*]

1 Department of Biochemistry, Molecular Biology B and Immunology, School of Medicine, University of Murcia, Regional Campus of International Excellence "Campus Mare Nostrum", 30100 Murcia, Spain
2 Obesity and Metabolism Laboratory, Biomedical Research Institute of Murcia (IMIB), 30120 Murcia, Spain
3 Radiopharmacy Research Group, Biomedical Research Institute of Murcia (IMIB), 30120 Murcia, Spain
4 Department of Biotechnology, Genomic and Plant Breeding, Murcian Institute for Agricultural and Environmental Research (IMIDA), 30150 Murcia, Spain
* Correspondence: bruno.ramos@imib.es (B.R.-M.); abel@um.es (A.A.L.-P.)
† These authors contributed equally to this work.

List of contents

Table S1. Calibration curves used for polyphenols quantification.

Table S2. Statistic parameters of silk fibroin nanoparticles precipitation reproducibility and repeatability of the precipitation

Table S3. Effect of irradiated SFN-PPh, non-irradiated SFN-PPh, and free compounds on cell viability of the human macrophage HL-60 differentiated cells.

Table S4. Effect of irradiated SFN-PPh, non-irradiated SFN-PPh, and free compounds on cytokine release (IL-6 and TNF-α) of the human macrophage HL-60 differentiated cells measured by ELISA.

Figure S1. Photographs showing the effect of SFN/PPh ratio in the PLC of CUR, Q and RES under white light and UV-light (λ=365nm). From left to right on each picture: SFN, PPh/SFN ratio 1:32, PPh/SFN ratio 1:16, PPh/SFN ratio 1:8, PPh/SFN ratio 1:4 and free PPh.

Figure S2. Photograph of the SFN-PPh stocks suspensions at 10 mg·mL^{-1} used for cell culture tests. From left to right in the picture: SFN, SFN-CUR, SFN-Q and SFN-RES.

Figure S3. FESEM images of the nanoparticles showing changes during sterilization by gamma irradiation. Scale bar= 400 nm.

Figure S4. ATR-FTIR Spectra of the prepared SFN-Q at different Q/SFN ratios: (a) Magnification of the region 3600-2600 cm^{-1} (b) Magnification of the region 950-900 cm^{-1}. (c) Peak resolution of the quercetin spectrum in the region 1600-600 cm^{-1}. (d) Peak resolution of the SFN spectrum in the region 1600-600 cm^{-1}.

Table S1. Calibration equations used for polyphenols quantification.

PPh	Wavelength (nm)	Equation	R^2
CUR	495	Abs = 3,6724·[CUR][a] + 0,0059	0.9986
Q	405	Abs = 11.278·[Q][a] - 0.0094	0.9924
RES	355	Abs = 2,319·[RES][a] + 0,0461	0,9949

[a]Concentration of polyphenols expressed in mg·mL^{-1}.

Table S2. Statistic parameters of silk fibroin nanoparticles precipitation reproducibility and repeatability of the precipitation.

**Parameter**	**Z_{ave} (d.nm)**	**PdI**	**ζ (mV)**
Number of values	18	18	18
Minimum	139.4	0.07300	-31.20
Maximum	149.3	0.14600	-23.60
Range	9.9	0.07300	7.60
10% Percentile	141.6	0.08020	-29.94
90% Percentile	148.9	0.13970	-24.23
Mean	145.1	0.11060	-27.71
Std. Deviation	2.8	0.01872	1.98
Std. Error of Mean	0.6	0.00441	0.46
Lower 95% CI of mean	143.7	0.10120	-28.70
Upper 95% CI of mean	146.5	0.11990	-26.73

Table S3. Effect of irradiated SFN-PPh, non-irradiated SFN-PPh, and free compounds on cell viability of the human macrophage HL-60 differentiated cells.

PPh	Concentration (μg·mL^{-1})	Irradiated SFN-PPh (%)	Non-irradiated SFN-PPh (%)	Free PPh (%)	p-value
CUR	16	106.00 ± 9.00	79.00 ± 3.00	84.00 ± 4.00	N.S.
	80	83.00 ± 3.00	67.00 ± 3.00	64.00 ± 3.00	N.S.
Q	16	106.00 ± 11.00	110.00 ± 4.00	109.00 ± 6.00	N.S.
	80	106.00 ± 7.00	114.00 ± 8.00	91.00 ± 4.00	N.S.
RES	16	109.00 ± 7.00	129.00 ± 2.00	87.00 ± 13.00	N.S.
	80	33.00 ± 6.00	23.00 ± 2.00	23.00 ± 2.00	N.S.

Data are presented as % of cell viability ± SE of LPS-stimulated (0.1 μg·mL^{-1}) HL-60 differentiated cells in the presence of irradiated or non-irradiated SFN-PPh (-CUR, -Q, and -RES) and free compounds (CUR, Q and RES) at different concentrations (16 and 80 μg·mL^{-1}) measured by MTT assays (n=3, three independent experiments performed in triplicate). Statistical differences were calculated using the two-way ANOVA test (considering $p<0.05$ significant), followed by a Dunnet's post hoc analysis for multiple comparisons. All data presented are normalized compared to untreated cells (100% viability). LPS, lipopolysaccharide; SFN, Silk fibroin nanoparticles; PPh, polyphenol; SFN-CUR, SFN-curcumin loaded; SFN-Q, SFN-quercetin loaded; SFN-RES, SFN-resveratrol loaded; CUR, curcumin; Q, quercetin; RES, resveratrol; N.S., not significant.

Table S4. Effect of irradiated SFN-PPh, non-irradiated SFN-PPh, and free compounds on cytokine release (IL-6 and TNF-α) of the human macrophage HL-60 differentiated cells measured by ELISA.

	IL-6				TNF-α			
	Irradiated SFN-PPh	Non-irradiated SFN-PPh	Free PPh	*p*-value	Irradiated SFN-PPh	Non-irradiated SFN-PPh	Free PPh	*p*-value
CUR	0.26 ± 0.04	0.15 ± 0.028	0.61 ± 0.13	N.S.	0.87 ± 0.03	0.73 ± 0.07	1.07 ± 0.11	N.S.
Q	0.17 ± 0.04	0.17 ± 0.04	0.18 ± 0.04	N.S.	1.08 ± 0.11	1.17 ± 0.09	0.88 ± 0.12	N.S.
RES	0.74 ± 0.05[a,b]	0.34 ± 0.02[b]	0.95 ± 0.10[a]	0.012	0.80 ± 0.18	0.58 ± 0.02	0.64 ± 0.06	N.S.

Values are expressed as fold-change compared to the untreated cells. Data are presented as mean ± SE of LPS-stimulated (0.1 μg·mL^{-1}) HL-60 differentiated cells in the presence of irradiated or non-irradiated SFN-PPh (-CUR, -Q, and -RES) and free compounds (CUR, Q and RES) at 16 μg·mL^{-1} measured by ELISA assays (n=3, three independent experiments performed in triplicate). Statistical differences were calculated using the two-way ANOVA test (considering $p<0.05$ significant), followed by Dunnet's post hoc analysis for multiple comparisons. LPS, Lipopolysaccharide; IL-6, Interleukin 6; TNF-*α*, tumor necrosis factor alpha; SFN, Silk fibroin nanoparticles; PPh, polyphenols; SFN-CUR, SFN-curcumin loaded; SFN-Q, SFN-quercetin loaded; SFN-RES, SFN-resveratrol loaded; CUR, curcumin; Q, quercetin; RES, resveratrol; N.S., not significant.

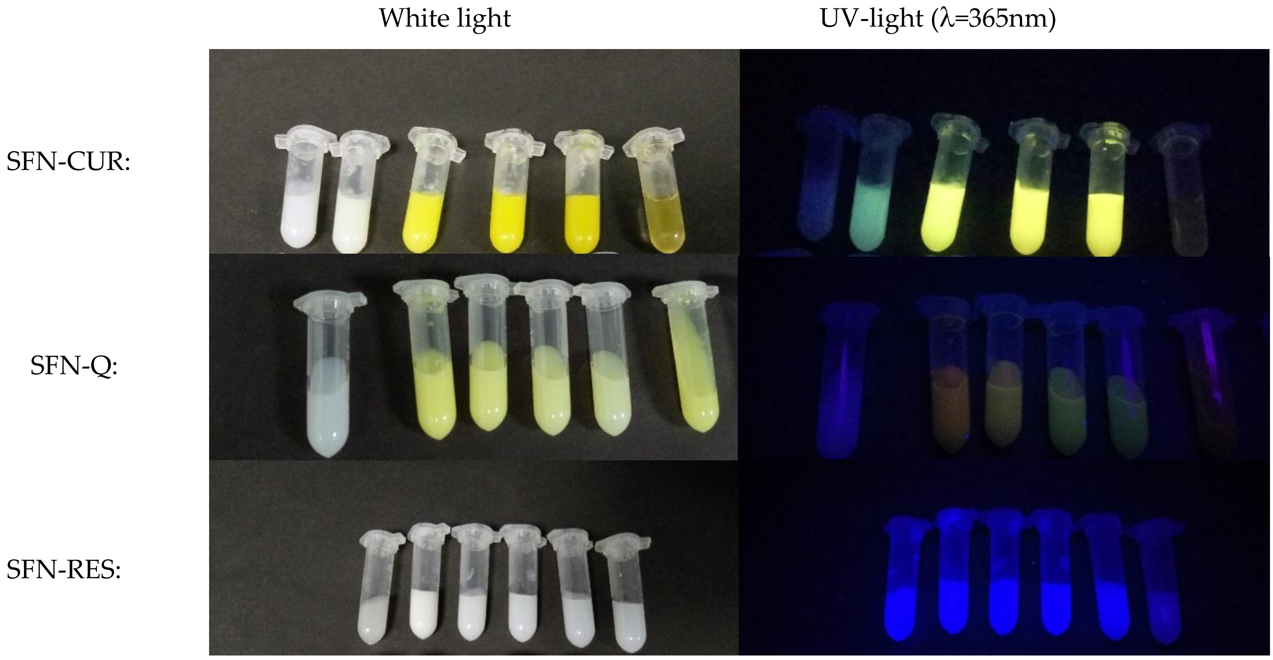

Figure S1. Photographs showing the effect of SFN/PPh ratio in the PLC of CUR, Q and RES under white light and UV-light (λ=365nm). From left to right on each picture: SFN, PPh/SFN ratio 1:32, PPh/SFN ratio 1:16, PPh/SFN ratio 1:8, PPh/SFN ratio 1:4 and PPh.

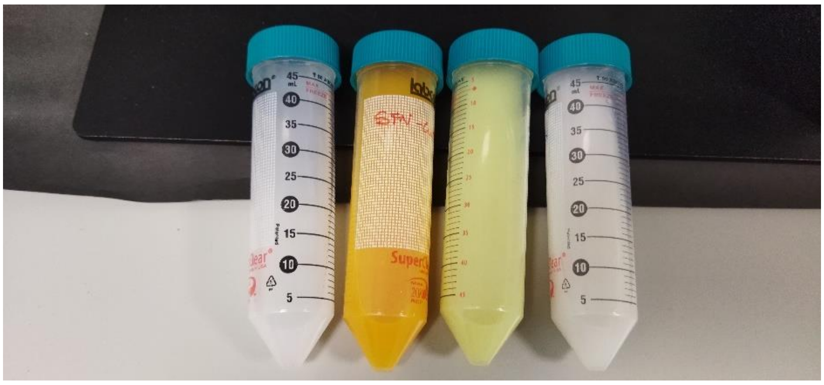

Figure S2. Photograph of the SFN-PPh stocks suspensions at 10 mg·mL^{-1} used for cell culture tests. From left to right in the picture: SFN, SFN-CUR, SFN-Q and SFN-RES.

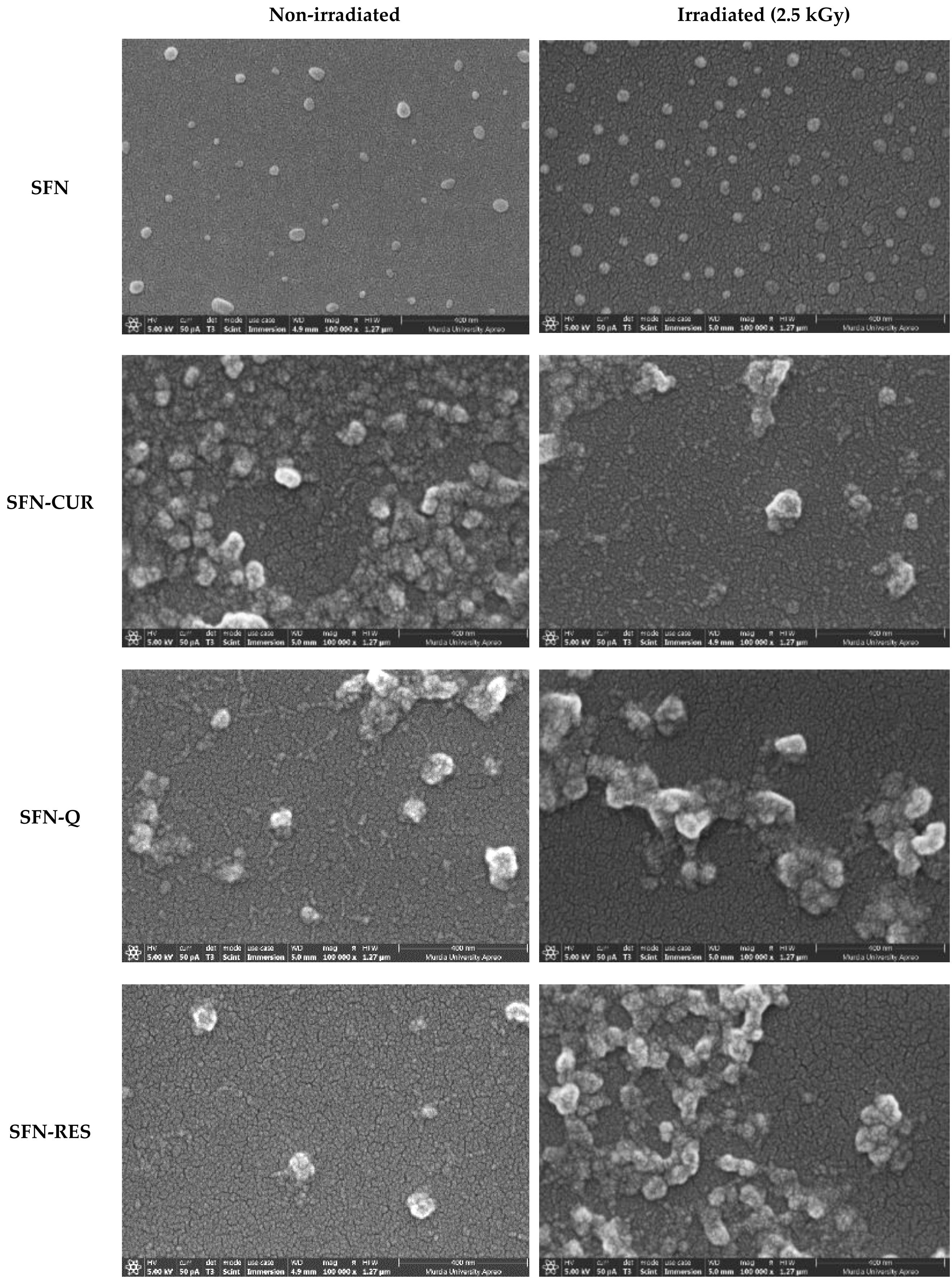

Figure S3. FESEM images of the nanoparticles showing changes during sterilization by gamma irradiation. Scale bar= 400 nm.

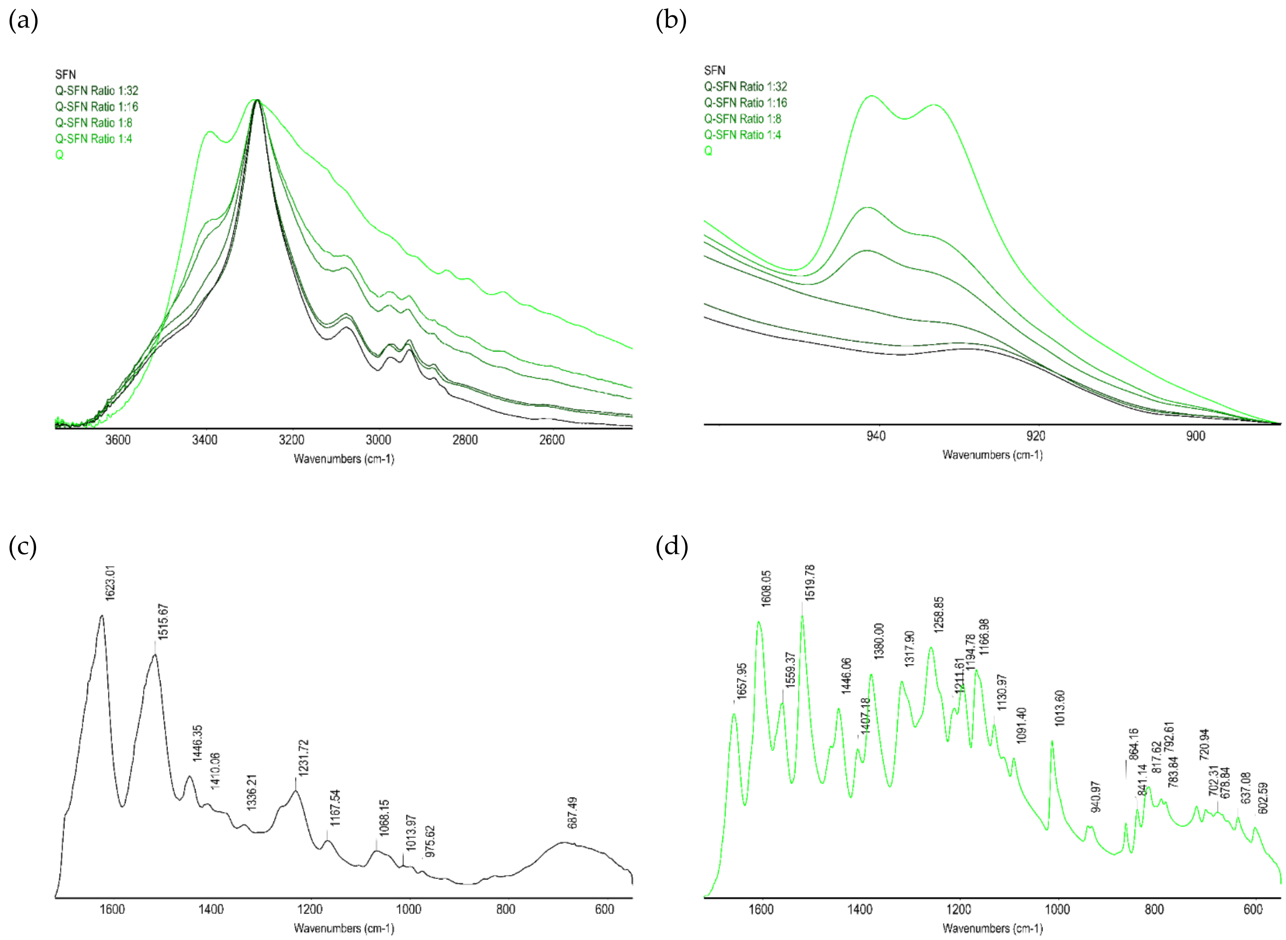

Figure S4. ATR-FTIR Spectra of the prepared SFN-Q at different Q/SFN ratios: (a) Magnification of the region 3600-2600 cm^{-1} (b) Magnification of the region 950-900 cm^{-1}. (c) Peak resolution of the quercetin spectrum in the region 1600-600 cm^{-1}. (d) Peak resolution of the SFN spectrum in the region 1600-600 cm^{-1}.
